# Reduced Left Ventricular Twist Early after Acute ST-Segment Elevation Myocardial Infarction as a Predictor of Left Ventricular Adverse Remodelling

**DOI:** 10.3390/diagnostics13182896

**Published:** 2023-09-09

**Authors:** Mihai-Andrei Lazăr, Ioana Ionac, Constantin-Tudor Luca, Lucian Petrescu, Cristina Vacarescu, Simina Crisan, Dan Gaiță, Dragos Cozma, Raluca Sosdean, Diana-Aurora Arnăutu, Alina-Ramona Cozlac, Slivia-Ana Luca, Andra Gurgu, Claudia Totorean, Cristian Mornos

**Affiliations:** 1 Cardiology Department, “Victor Babes” University of Medicine and Pharmacy, 2 Eftimie Murgu Sq., 300041 Timisoara, Romania; mihai88us@yahoo.com (M.-A.L.); petrescu_lucian@yahoo.com (L.P.); cristina.vacarescu@umft.ro (C.V.); urseanusimina@yahoo.com (S.C.); dgaita@cardiologie.ro (D.G.); dragoscozma@gmail.com (D.C.); ralusosdean@yahoo.com (R.S.); nicoramo_alina@yahoo.com (A.-R.C.); silvia.luca0@yahoo.com (S.-A.L.); gurgu_andra@yahoo.com (A.G.); claudia_hudrea@yahoo.com (C.T.); mornoscristi@yahoo.com (C.M.); 2 Institute of Cardiovascular Diseases Timisoara, 13A Gheorghe Adam Street, 300310 Timisoara, Romania; bordejevic.aurora@gmail.com; 3 Research Center of the Institute of Cardiovascular Diseases Timisoara, 13A Gheorghe Adam Street, 300310 Timisoara, Romania

**Keywords:** ST-elevation myocardial infarction (STEMI), left ventricular remodelling, speckle-tracking echocardiography, myocardial strain, left ventricular layers, twist

## Abstract

Background: The left ventricular (LV) remodelling process represents the main cause of heart failure after a ST-segment elevation myocardial infarction (STEMI). Speckle-tracking echocardiography (STE) can detect early deformation impairment, while also predicting LV remodelling during follow-up. The aim of this study was to investigate the STE parameters in predicting cardiac remodelling following a percutaneous coronary intervention (PCI) in STEMI patients. Methods: The study population consisted of 60 patients with acute STEMI and no history of prior myocardial infarction treated with PCI. The patients were assessed both by conventional transthoracic and ST echocardiography in the first 12 h after admission and 6 months after the acute phase. Adverse remodelling was defined as an increase in LVEDV and/or LVESV by 15%. Results: Adverse remodelling occurred in 26 patients (43.33%). By multivariate regression equation, the risk of adverse remodelling increases with age (by 1.1-fold), triglyceride level (by 1.009-fold), and midmyocardial radial strain (mid-RS) (1.06-fold). Increased initial twist decreases the chances of adverse remodelling (0.847-fold). The LV twist presented the largest area under the receiver operating characteristic (ROC) curve to predict adverse remodelling (AUROC = 0.648; 95% CI [0.506;0.789], *p* = 0.04). A twist value higher than 11° has a 76.9% specificity and a 72.7% positive predictive value for reverse remodelling at 6 months.

## 1. Introduction

Worldwide, coronary artery disease is the leading cause of mortality, and its prevalence is recognised as an upward trend. Nevertheless, in Europe, in the last 30 years, there has been a tendency for a decrease in ischaemic heart disease mortality [1]. Furthermore, the relative incidences of ST-segment elevation myocardial infarction (STEMI) and non-ST-segment elevation myocardial infarction (NSTEMI) are decreasing and increasing, respectively [2,3]. There is a directly proportional relationship between the lower mortality in STEMI and the wider use of reperfusion therapy, primary percutaneous coronary intervention (PCI) and new antithrombotic drugs, and secondary prevention [4].

Heart failure (HF) is both the most common complication and also the most important prognostic element in patients with STEMI [5,6]. The pathophysiology of HF in patients with STEMI is based on a complex, continuous, molecular, and cellular alteration called “ventricular remodelling” (REM). It was first described by Tennant and Wiggers and involves dilation of the ventricle, the formation and expansion of the scar, and geometrical rearrangement of the normal structures of the heart, including a more spherical shape of the left ventricle (LV), with the proliferation of the extracellular matrix being one of the hallmarks of pathological REM [7,8]. Up to a point, REM is seen as an adaptative and compensatory process, but soon becomes pathological and leads to HF [8]. Arbitrarily, LV remodelling recognizes two forms: adverse and reverse remodelling, and it is defined as the changes in LV end-diastolic volume (LVEDV) and/or end-systolic volume (LVESV) from the first in-hospital to the follow-up measurements [9,10]. Adverse remodelling is characterised by an increase in LVEDV, while reverse remodelling is defined as a considerable decrease in LVESV, LV ejection fraction (LVEF) augmentation, or wall motion score index improvement.

The LV myocardium consists of three layers, and there is a difference in transmural damage after a myocardial infarction (MI). The ischaemic process begins in the endocardium and progresses as a wave front to the epicardium, the first layer being the most-affected [11].

Over the years, the role of echocardiography in the evaluation of patients with MI has been crucial. As echocardiographic techniques have improved, it has been possible to develop new methods, such as speckle-tracking echocardiography. Moreover, the study of individual myocardial deformation at the level of the three myocardial layers is a promising tool in the study of LV function.

The aim of this study was to identify the echocardiographic parameters with high sensitivity and specificity for early identification of the progression of REM after STEMI. We sought to assess the alteration in LV rotational characteristics by regional myocardial wall motion abnormalities after STEMI. Moreover, we hypothesised that the difference between the myocardial layers and the combination of different parameters obtained from speckle-tracking echocardiography could be important prognostic factors to predict REM. Differences in layer-specific strain values could be explained by human heart distribution of fibres that present various angles. Fibre distribution has angles varying from about 60° (in a circumferential plane) at the inner surface to about–60° at the outer surface. The ratio of circumferentially to longitudinally oriented fibres is 10:1, increasing from the apex to the base [12]. Therefore, layer-specific strain could be a noninvasive tool to rapidly identify patients with suspected acute coronary syndromes who would benefit from reperfusion therapy [13].

A crucial aspect of the LV function is cardiac rotation during systole. A wringing action is produced by the LV muscle fibres that are angled from a right-hand helix in the subendocardium, to a left-hand helix in the subepicardium [14,15]. When observed from the apex during systole in healthy individuals, the apex rotates counterclockwise, while the base rotates clockwise. The LV twist, which is the net rotational difference between the LV apex and base, determines a uniform distribution of LV fibre stress and fibre shortening across the wall. It has been demonstrated that in both animals and people, a reduction in the LV twist, which is related to global ventricular performance, is caused by acute myocardial ischaemia and infarction [16,17,18].

In patients with anterior wall MI, the apical peak circumferential strain is severely reduced when associating LV systolic dysfunction compared with those with preserved systolic function [19]. Furthermore, the LV twist is significantly depressed in patients who have reduced LVEF, mostly due to the reduced magnitude of LV apical rotation. In addition to this, as systolic dysfunction progresses, diastolic untwisting is also reduced and delayed [18]. However, there has been little data about the impact of acute MI on the LV twist (T).

Mechanical dispersion (MD) represents a marker derived from 2D STE (2D speckle-tracking echocardiography) and reflects contraction heterogeneity. In normal circumstances, all myocardial segments have broadly similar contraction durations; therefore, the values for MD are normally low. Patients with ventricular arrhythmias and ischaemic and nonischaemic cardiomyopathies have higher values of MD than those that did not present ventricular arrhythmias [20]. In addition to that, longer MD was associated with a worse prognosis in patients with STEMI [21].

## 2. Materials and Methods

### 2.1. Study Population

We prospectively analysed 200 patients with STEMI and PCI (successful PCI was defined as residual stenosis <20%) in sinus rhythm, hospitalised at the Cardiology Department of Timisoara Institute of Cardiovascular Diseases, in the period of December 2018 to December 2021. We excluded patients with inadequate echocardiographic images that impeded accurate measurements, open-chest surgery, previous myocardial infarction, patients with a cardiac pacemaker and/or implantable cardioverter defibrillator, malignant neoplasia, and those with a life expectancy <1 year. The remaining 151 patients formed our study population. Unfortunately, due to the problems caused by the COVID-19 pandemic, we could not re-evaluate all of these patients, and the final cohort consisted of 60 patients. At follow-up, the patients were divided into two groups: patients who suffered adverse remodelling and patients with reverse remodelling. Those who did not fit into any category were defined as normal (no remodelling).

Patients were treated with beta-blockers, angiotensin-converting enzyme inhibitor (ACEI), angiotensin receptor II blocker (ARB), spironolactone, statins, and dual antiplatelet therapy, according to current European guidelines. The study was conducted according to the guidelines of the Declaration of Helsinki and approved by the Institutional Ethics Committee of Institute of Cardiovascular Diseases Timisoara (8461/04.12.2018). All patients provided written informed consent for participation in the study.

### 2.2. Clinical and Humoral Variables

The following clinical variables were recorded at hospital discharge: age, sex, body mass index, systolic arterial pressure, and heart rate. Furthermore, high-sensitivity cardiac troponin I level, time of peak CK, thrombolytic therapy, and TIMI flow before and after PCI were recorded. For study purposes, five cardiovascular risk factors were considered as follows: hypertension (systolic blood pressure >140 mmHg, diastolic blood pressure >90 mm Hg, and in drug treatment), cardiovascular disease heredity, smoking (≥1 cigarette/day, cessation of smoking <10 years previously was still considered as smoking), diabetes mellitus (fasting glycemia >126 mg/dL or in drug treatment), and hypercholesterolemia (>200 mg/dL or in drug treatment).

### 2.3. Ecocardiography

The patients underwent a transthoracic echocardiography in the first 12 h after admission, at rest, in the left lateral decubitus position. All the echocardiograms were performed before myocardial revascularisation. Both conventional two-dimensional echocardiography and speckle-tracking echocardiography were performed at baseline and follow-up. All measurements were performed by an experienced echocardiographer with an ultrasonographic system (Vivid 9 General Electric, Milwaukee, WI, USA) equipped with a multifrequency transducer. LV ejection fraction (LVEF) was calculated using the modified Simpson’s biplane method, in apical two- and four-chamber views [22]. These measurements were used to define the two types of cardiac remodelling: adverse and reverse. Adverse remodelling is defined as an increase in LVEDV and/or LVESV by 15%. By contrast, reverse remodelling with myocardial recovery is defined as an improvement in wall motion score index or increase in LVEF greater than 5% or decrease in LVESV greater than 15%.

The left atrial (LA) volume was also determined by planimetry, using the biplane method, and indexed to the body surface. Systolic pulmonary artery pressure (SPAS) was calculated using the tricuspid regurgitation flow pressure and the right atrial pressure, estimated according to the inferior vena cava dimensions and respiratory variability. The mitral flow patterns were recorded from the apical four-chamber view using a 4–5 mm pulsed Doppler sample volume placed between the mitral leaflets’ tips, in diastole. Five consecutive cardiac cycles were recorded, with a horizontal sweep of 100 mm/s. The peak velocities of early diastolic mitral flow (E wave), and late diastolic mitral flow (A wave) were measured during end-expiratory apnoea. The results were averaged over the velocities recorded for the five consecutive cardiac cycles.

For TDI parameter evaluation and measurement (m/s)—peak systolic tricuspid annulus velocity (tricuspid annulus s’), peak systolic mitral annulus velocity (mitral annulus s’), and peak early (e’) and late (a’) diastolic mitral annular velocity, the TDI program was set in pulsed-wave Doppler mode [23] with a 4–5 mm sample volume. For the tricuspid valve annulus, the sample volume was placed at the site of the attachment of the anterior leaflet, whereas for the mitral valve annulus, it was sequentially placed at the septal and lateral wall sites. For the last value, the values were considered both separately and averaged between the septal and lateral measurements. Also, for all these parameters, the values were averaged on five consecutive cardiac cycles. E/e’ and E/(e’ × s’) ratios were calculated using the resulting values. All measurements were repeated six months after hospital discharge.

Myocardial contractility was categorised into four grades: normokinesia, hypokinesia, akinesia, and dyskinesia.

To assess both the three types of strains (longitudinal, circumferential, and radial) and the layer-specific strain, the apical four-chamber and two-chamber views and long-axis and short-axis views (at the level of mitral valve, papillary muscles, and heart apex) were recorded with a frame rate of 60–80 frame/s during baseline and follow-up echocardiographic evaluations. While acquiring images, the LV basal short-axis view was identified by the presence of mitral leaflets, while excluding the mitral annulus, and the apical view was identified by the presence of a LV cavity in the absence of papillary muscles. We made every effort to obtain the LV cross-section as circular as possible. Then, strain analysis was performed with the off-line software (EchoPAC PC, GE Medical Systems). After manual tracing of the endocardial border of the LV in three apical images, endocardial, midmyocardial, and epicardial segmental and global longitudinal strains (GLSs) were obtained using LSendo, LSmid, and LSepi, respectively. Basal and apical LV radial and circumferential peak strains (RS and CS, respectively) were defined as the mean strain of the six basal and four apical parasternal short-axis segments. Rotation (degrees) was obtained at the basal level and the LV apex. The ratio between the endocardium and epicardium was also calculated according to the difference between LSendo and LSepi. LV twist was defined as the net difference (in degrees) of apical and basal rotations at isochronal time points and was autocomputed by the software from the values of the basal and apical rotations. A pulse-wave Doppler tracing obtained from the LV outflow tract was used to identify the timing of aortic valve opening and closure. In order to verify the inter- and intraobserver variability speckle-tracking measurements, they were performed by one observer at two separate time points and by two investigators that were unaware of one another’s measurements and of the study time point, in a group of 30 randomly selected subjects.

### 2.4. Statistical Analysis

Continuous variables were presented as a mean with standard deviation (SD) or as a median with an interquartile range (IQR) and categorical variables, such as frequency and percentages. Considering that the results of the normality test (Shapiro–Wilk) showed a non-Gaussian distribution, we used further nonparametric tests for our analysis. The Wilcoxon test was employed to compare the patient’s initial characteristics and after twelve months. A Kruskal–Wallis test, followed by a post hoc analysis with Mann–Whitney U test characteristics, was conducted with a Bonferroni correction to compare the patients’ characteristics regarding cardiac remodelling. Several multivariate regression models were built using the Akaike criterion to assess the independent factors for cardiac remodelling. In the final regression equations, the predictors were accepted according to a repeated backward-stepwise algorithm (inclusion criteria *p* < 0.05, exclusion criteria *p* > 0.10) to obtain the most appropriate theoretical model to fit the collected data. Regression models were built using a training test partition. The training was performed on a randomly selected partition consisting of 80% of our data, while the testing was performed on the remaining 20%. Training and testing of the classifiers were carried out by a repeated 10-fold cross-validation method. To avoid biased predictions, we averaged model performance metrics across test folds. A characteristic (ROC) curve was employed to illustrate the identification ability, and the thresholds to discriminate between future adverse remodelling and normal remodelling were determined by Youden’s index. Data analysis was performed using R: A Language and Environment for Statistical Computing (R Core Team, Vienna, Austria). A value of *p* under 0.05 was considered to indicate statistically significant differences.

## 3. Results

### 3.1. Patients’ Characterisitics

The patients’ baseline characteristics are presented in Table 1A,B. A total of 25% (n = 15) of the patients were diabetic, 73.3% (n = 44) were hypertensive, 66.7% (n = 40) were smokers, and all the study population had abnormal lipid profiles. A total of 35 patients (58.3%) underwent primary percutaneous coronary intervention, whereas 25 patients (41.7%) were subjected to a pharmacoinvasive strategy. Most patients had an anterior STEMI (n = 30; 50%) and complete revascularisation (n = 36; 60.0%). The TIMI flow was 3, both before PCI (n = 32; 53.3%) and after PCI (n = 53; 88.3%). For the most part, the CK peak occurred at 6 h after admission (n = 38; 63.3%). Primary PCI was performed in the first 12 h after initial chest pain in all patients.

Baseline and follow-up echocardiographic parameters are presented in Table 2. At follow-up, after 6 months, there was a significant difference in the LV end-diastolic diameter (*p* < 0.001) and LV end-diastolic volume (*p* = 0.033), with both of the values being higher. As far as the right ventricular systolic function is concerned, both the RVFW s’ and TAPSE increased in 6 months (0.15 m/s, 24 mm, respectively; *p* = 0.036 for RVFW s’ and *p* = 0.002 for TAPSE).

The results of 2D speckle-tracking echocardiography (STE) at baseline and after 6 months are shown in Table 3.

The median LVEF was 46.00% (40.75, 51.00), and the GLS was −13.25% (−14.57, −11.55) at baseline. On follow-up, these two parameters significantly increased: the LVEF was 50.00% (41.75, 53.00) (*p* = 0.018), and the global longitudinal strain was −15.60% (−18.25, −12.38) (*p* < 0.001).

**Figure 1:**
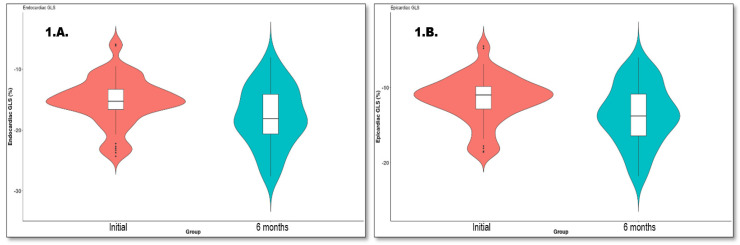
(**A**) L.S endo evolution and (**B**) L.S epi evolution.

**Figure 2:**
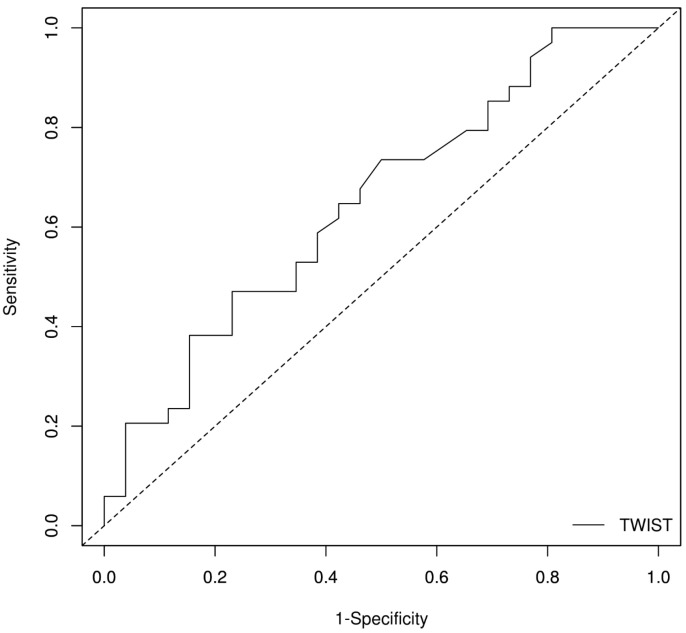
Receiver operating characteristic (ROC) curve for twist to predict adverse remodelling.

On initial evaluation, the longitudinal strain (LS) increased from the epicardial (LS epi) layer to the endocardial (LS endo) layer (LSendo and LSepi were −15.15 (−16.50, −13.20) and −11.00 (−12.72, −9.80), respectively). This pattern was also observed at 6 months when LS endo was −18.10 (−20.62, −14.10) (*p* < 0.001) and LS epi was −13.80 (−16.45, −10.88) (*p* < 0.001). Furthermore, we assessed the LS endo/epi ratio, with no significant differences between baseline and follow-up (1.33 (1.27, 1.37) at admission and 1.30 (1.25, 1.36) at follow-up, *p* = 0.496). On the other hand, there was no difference in the mid-CS endo, mid-CS epi, and mid-CS endo/epi ratio at follow-up (mid-CS endo: −18.50 (−22.30, −14.05), −19.60 (−25.00, −13.50); mid-CS epi: −7.00 (−10.20, −5.70), −8.00 (−11.05, −6.40); and mid-CS endo/epi ratio: 2.45 (1.80, 2.83), 2.23 (1.52, 2.79)). Figure 1 and Figure 2 show the evolution of the LS endo and the LS epi.

In addition, there were greater values at 6 months of the following parameters: base CS, apical CS, and apical RS (at inclusion: −10.80 (−13.40, −6.40), −11.70 (−16.40, −8.80), and 21.00 (13.00, 31.20), respectively, and −11.40 (−14.20, −9.40), −13.20 (−18.45, −9.50), and 24.33 (16.10, 40.05), respectively, at 6 months) (*p* = 0.004, *p* = 0.033, and 0.009).

Multivariate linear regression analysis was used to evaluate the independent predictor factors for LS endo at follow-up. Our results highlight that the initial values of LVEF and e/e ratio, among diabetics, those who received thrombolytic therapy and ACEI/ARB treatment explained 61.1% of the LS endo variance (R^2^ = 0.611) (Table 4).

### 3.2. Cardiac Remodelling

Remodelling was classified as reverse (n = 13, 21.67%) and adverse (n = 26, 43.33%), and the patients who did not fit into any category were defined as being normal (no remodelling) (n = 21, 35%).

The baseline demographic, clinical, and biochemical characteristics of the three STEMI patient groups are compared in Table 5. Patients with adverse remodelling had a higher incidence of hypertension (*p* = 0.035). Moreover, there was no difference regarding the distribution of age, gender, smoking, diabetes mellitus, and obesity among the three groups.

The laboratory and angiographic characteristics in cardiac remodelling groups are listed in Table 6. Patients with adverse remodelling had a smaller body surface area and a higher incidence of anterior myocardial infarction and circumflex artery as the culprit lesion.

The general echocardiographic parameters are listed in Table 7. The only parameter whose change was statistically significant was the LVEDV (*p* < 0.001).

In terms of speckle-tracking echocardiography, mechanical dispersion and radial strain at the level of papillary muscles (mid-RS) are marginally significant between the adverse and reverse remodelling groups (*p* = 0.073 and *p* = 0.064, respectively). The results are labelled in Table 8.

In order to assess the independent factors that predict the risk of developing adverse remodelling in our study population, we employed a backward multivariate logistic regression model. Akaike information criteria (AIC) were used in order to determine the best model. The odds ratio and a 95% confidence interval were calculated. Our regression equation proved to be a good fit for the model, explaining 36.2% of the adverse remodelling (R^2^ = 0.286). The risk of adverse remodelling increases with age (by 1.1-fold), triglycerides (by 1.009-fold), and mid-RS (1.06-fold). An increased initial twist decreases the chances of adverse remodelling (0.847-fold) (Table 9).

The multivariate logistic regression of independent risk factors for adverse remodelling showed us that the initial value of twist has a high impact when it comes to predicting the adverse remodelling at 6 months. For this reason, we employed a ROC analysis in order to find the threshold point that predicts adverse remodelling (Figure 2).

The AUROC of the twist for adverse remodelling was 0.648; 95% CI [0.506;0.789], *p* = 0.04 (Table 10). The Youden index indicates an optimal cut-off value of 11° for the twist (Table 11). A twist value higher than 11° has a 76.9% specificity and a 72.7% positive predictive value for normal remodelling at 6 months.

## 4. Discussion

To the best of our knowledge, this is the first study that evaluated the twist as a predictor of cardiac remodelling after STEMI. Predicting prognosis in STEMI patients has grown in clinical significance. In patients with STEMI, the rapid recognition of high-risk patients who may benefit from an intensified treatment strategy is of major importance [24,25,26].

Contrary to what is observed in the literature [27], with the exception of hypertension, in our study, there was no difference regarding the distribution of age, gender, and cardiovascular risk factors among the groups with reverse and adverse cardiac remodelling.

Regional and global myocardial functions are determined by myocardial strain; that is an important echocardiographic tool. GLS has been validated to evaluate global cardiac function in the setting of STEMI, stable chronic coronary artery disease, and general healthy populations [28,29,30,31].

Because LVEF does not accurately evaluate intrinsic myocardial deformation, it is possible for it to be normal even when the LV systolic function is compromised. Moreover, in the studied group, the LVEF did not register a statistically significant difference in the adverse remodelling group compared to the reverse one (46% vs. 47%, *p* = 0.959).

The LV global longitudinal strain (GLS) evaluated by speckle-tracking echocardiography may overcome these restrictions, and it has been proven to be a crucial prognostic parameter in the risk stratification of patients following AMI.

Based on previous studies, there is still no consensus on the GLS cut-off value that most accurately predicts mortality and morbidity. Studies have shown that GLS is below 12–15% in most cases. Bendery et al. recently suggested that a baseline GLS score <12.65% was the only significant independent predictor of 30-day MACE, with a sensitivity of 77.8% and a specificity of 83.7% [32]. Guedemans et al. established a threshold of 14.4%, and less than this was suggested to be significantly correlated with all-cause mortality in chronic obstructive pulmonary disease (COPD) patients after STEMI [33]. In the two groups of cardiac remodelling, the GLS had very similar values (−13.25% in the adverse group and −13.2% in the reverse remodelling one (*p* = 0.587)), which are superimposed over the ones in the above studies.

Myocardial injury caused by ischaemia begins from the endocardium and spreads to the myocardium and epicardium as a result of the epicardial location of the coronary arteries and LV systolic pressure gradient during systole, which is called the wave-front phenomenon [34]. Therefore, the assessment of both myocardial transmural ischaemia and endocardial damage is crucial in determining the prognosis of coronary artery disease.

Skaarup et al., in a study that included [27] 465 patients who suffered from acute coronary syndrome (STEMI, non-STEMI, and unstable angina), showed that LV GLS measured at all layers was associated with adverse events (heart failure and cardiovascular death). Furthermore, only LV GLS and LV GLS displayed at the epicardium were highly associated with adverse events. Moreover, only LV GLS at the epicardium was independently correlated with cardiovascular mortality.

In agreement with our study, both Hamada et al. [35] and Skaarup et al. [27] found a gradient across the LV myocardial wall that decreases from the endocardium to the epicardium. We hypothesised that the ratio between the endocardium and epicardium strain gradient could be indicative of the myocardial remodelling process (LS endo/epi ratio). The explanation for why this ratio did not predict the phenomenon of cardiac remodelling could be represented by the fact that both the strain at the endocardial level and the one at the epicardial level register proportional changes, with the ratio being constant. 

Song et al. examined the longitudinal, circumferential, and radial LV strains in a group of patients undergoing PCI after inferior STEMI. Following PCI, they showed a 60% improvement in global longitudinal and circumferential LV stresses and a lower LV strain (in all three dimensions) compared to healthy controls. In patients with inferior MI, the LV regional strain was impaired in 87% of cases and it improved in 60% of cases following PCI [36].

Lower, in 1669, was the first to describe the twisting motion of the LV as “the wringing of a linen cloth to squeeze out the water” [37]. Over the past three centuries, the twist deformation of the LV has continued to intrigue clinicians and researchers in their quest to understand the performance of the human heart. The LV twist or torsion represents the mean longitudinal gradient of the net difference in clockwise and counterclockwise rotations of the LV apex and base, as viewed from the LV apex.

It has been reported that acute myocardial ischaemia and infarction are responsible for a decrease in the LV twist that is related to global ventricular function in animals and humans [16,17,38]. There has been little data about the impact of AMI on LV rotation and twist. One of the main purposes of this study was to assess the alteration in LV rotational characteristics by regional myocardial wall motion abnormalities after STEMI. Cardiac rotation around the long axis is an important component of LV systolic function. The LV twist is related to myocardial contractility and structure and has recently been recognised as a sensitive indicator of cardiac performance. It has been shown that myocardial ischaemia and MI affect the transmural balance of LV torsional movements, thereby disturbing the LV systolic performance [38,39,40].

The base may rotate less than the apex and may have a reserve to rotate more to compensate, because the LV basal radius is greater than the LV apical radius. Additionally, the basal rotation can be enhanced and the akinetic apical wall abolished by the opposing power. Second, it is well recognised that anterior AMI typically results in the hypercontraction of the LV basal walls. In anterior MI, the normally perfused residual myocardium next to the coronary blockage exhibits compensatory hyperkinesia [41,42].

Outcomes in management of STEMI have dramatically improved in recent years. This is mainly due to the development of reperfusion therapy and modern pharmacotherapy, such as ACEI/ARB, beta-blockers, and spironolactone. The goal would be to establish an “individual pathogenic profile” and identify specific genetic variation-based therapies. Until novel drug treatment strategies are implemented, we should focus on primary and secondary prevention, and echocardiography is a major tool for early diagnosis [43]. In this current work, the risk of adverse remodelling increases with age (by 1.1-fold), triglyceride level (by 1.009-fold), and radial strain at the level of papillary muscles (mid-RS) (1.06-fold). An increased initial twist decreases the chances of adverse remodelling (0.847-fold).

As mentioned above, it was shown that the initial value of the twist has a high impact on predicting the adverse remodelling at 6 months. For this reason, we employed ROC analysis in order to find the threshold point that predicts adverse remodelling. The AUROC of the twist for adverse remodelling was 0.648; 95%CI [0.506; 0.789], *p* = 0.04; the Youden index indicates an optimal cut-off value of 11 for the twist. A twist value higher than 11 has a 76.9% specificity and a 72.7% positive predictive value for normal remodelling at 6 months.

Spinelli et al. [44] analysed 75 patients with anterior wall STEMI before and after myocardial revascularisation and at a 6-month follow-up. They sought to identify the impact of global LV torsion on reverse remodelling after STEMI, and they found that there was significant reduction in LV torsion early before revascularisation. Furthermore, the LV torsion significantly improved after PCI especially in patients who showed reverse LV remodelling in the follow-up. They concluded that improvement in global LV torsion following coronary artery revascularisation is the major predictor of reverse LV remodelling; 1.34°/cm for LV torsion after revascularisation (sensitivity 88% and specificity 80%) was the optimal cut-off value in predicting reverse remodelling of LV.

There was agreement between our results and the results of a study published by Bertini and his colleagues; they studied 66 patients with AMI, in addition to thirty age-matched healthy subjects as a control group. Their aim was to evaluate the impact of AMI on LV rotational. They found that, compared to normal, the LV twist was reduced in all AMI patients [40]. Furthermore, the LV twist and torsion might be good parameters to monitor the recovery of global longitudinal function [45]. Nevertheless, global LV torsion was decreased in acute STEMI patients soon after reperfusion and it showed marked improvement in LV torsion/twist. LV torsion early after reperfusion can predict LV remodelling at the 3-month follow-up [46]. Future studies may assess these rotational parameters after the acute phase, taking into consideration stress echocardiography, as little is known about rotational mechanics during dobutamine stress echocardiography [47].

## 5. Conclusions

In conclusion, our findings support the fact that 2D STE could be used in patients with STEMI who have undergone successful PCI. The LV twist at baseline predicts the occurrence of LV adverse remodelling at the 6-month follow-up. The LV twist cut-off value that was a predictor of worsening in LV function was 11°. Our study suggests that early twist is a widely available and practical parameter in predicting future left ventricular remodelling after an acute coronary event. This result may have important implications in stratifying risk in patients with STEMI. As judicious use of diagnostic and therapeutic modalities may prevent and/or reverse REM and improve quality of life and life span, the LV twist should be considered as a complementary approach in all patients early after MI.

## 6. Limitations

The most important limitation of this study is its small sample size. Further investigations are thus required to confirm our findings. This could also be the reason why we did not find any independent risk factor for reverse remodelling. Moreover, we did not assess the left ventricle with other imaging modalities, such as cardiac magnetic resonance imaging. All study subjects averaged six decades in age, and the results may be different from younger or older subjects, because it is known that LV rotational characteristics differ with age. There is a need for further research identifying potential mechanistic pathways and optimal patient selection criteria for available therapies promoting reverse remodelling and myocardial recovery.

Future directions should take into consideration the presence of a subendocardial-to-subepicardial gradient in LV mechanics that may provide a useful clinical measure for early recognition of a subclinical state that is likely to progress into either systolic or diastolic heart failure.

## Figures and Tables

**Table 1:** (**A**) Baseline characteristics of the study group: demographic data and biological parameters. (**B**) Baseline characteristics of the study group: angiographic data and discharge treatment.

(**A**)	
**Characteristic**	**Overall (n = 60)**
Age (years) Median (IQR)	57.50 (50.00, 66.00)
Male sex, (n, %)	43 (71.7%)
Smoking history, (n, %)	40 (66.7%)
Hypertension, (n, %)	44 (73.3%)
Diabetes mellitus, (n, %)	15 (25.0%)
Hypercholesterolemia, (n, %)	60 (100.0%)
Obesity, (n, %) Body mass index, kg/m^2^	15 (25.0%) 26.91 (25.38, 30.43)
Haemoglobin, (g/dL) Median (IQR)	14.55 (13.25, 15.43)
Glycemia, (g/dL) Median (IQR)	117.00 (108.00, 153.00)
White blood cells, (/microl) Median (IQR)	11055.00 (9135.00, 13225.00)
Creatinine, (mg/dL) Median (IQR)	1.03 (0.91, 1.16)
LDLc, (mg/dL) Median (IQR)	111.00 (94.25, 132.25)
TG, (mg/dL) Median (IQR)	145.00 (100.75, 197.50)
High-sensitivity cardiac troponin I, ng/L Median (IQR)	7122.50 (1304.00, 33542.75)
Peak CK	
At admission	22 (36.7%)
6 h after admission	38 (63.3%)
(**B**)	
Thrombolytic therapy	25 (41.7%)
Thrombolytic therapy effectiveness Myocardial infarction wall	21/25 (84.0%)
Anterior	30 (50.0%)
Inferior	24 (40.0%)
Other localisations	6 (10.0%)
TIMI flow before PCI	
0	22 (36.7%)
1	2 (3.3%)
2	4 (6.7%)
3	32 (53.3%)
TIMI flow after PCI	
0	3 (5.0%)
1	2 (3.3%)
2	2 (3.3%)
3	53 (88.3%)
BMS	
1	10 (16.7%)
2	2 (3.3%)
3	1 (1.7%)
DES	
1	28 (46.7%)
2	8 (13.3%)
3	2 (3.3%)
4	1 (1.7%)
Complete revascularisation, n (%)	36 (60.0%)
Treatment at discharge	
Beta-blockers, n (%)	48 (80.0%)
Spironolactone	49 (81.7%)
ACEI/ARB	42 (70.0%)

IQR: interquartile range; LDLc: low-density lipoprotein cholesterol; TG: triglycerides; CK: creatin kinase; TIMI: thrombolysis in myocardial infarction; PCI: percutaneous coronary intervention; BMS: bare metal stent; DES: drug-eluting stent; ACEI: angiotensin-converting enzyme inhibitor; ARB: angiotensin II receptor blocker. For numerical values in parentheses, the data are expressed by median and IQR (interquartile range).

**Table 2:** Echocardiographic parameters of the study population at baseline and at follow-up.

Variable	Initial (n = 60) Median (IQR)	6 Months (n = 60) Median (IQR)	*p* Value
E, m/s	0.69 (0.57, 0.82)	0.70 (0.62, 0.90)	0.056
A, m/s	0.76 (0.61, 0.92)	0.80 (0.60, 0.90)	0.629
E/A ratio	0.86 (0.71, 1.17)	0.92 (0.78, 1.37)	0.852
Mean e’, m/s	0.08 (0.07, 0.10)	0.09 (0.07, 0.10)	0.149
E/e’ ratio	8.00 (6.85, 10.00)	8.44 (6.65, 10.92)	0.651
Mean s’, m/s	0.08 (0.07, 0.09)	0.07 (0.07, 0.09)	0.311
E/(e’×s’) ratio	98.63 (81.09, 141.30)	102.94 (85.85, 157.92)	0.762
RVFW s’, m/s	0.14 (0.13, 0.16)	0.15 (0.13, 0.17)	0.036 *
Left atrial diameter, cm	4.00 (3.60, 4.15)	4.00 (3.70, 4.40)	0.007 *
Left atrial volume, mL	52.00 (40.00, 62.25)	54.00 (40.00, 70.00)	0.088
Indexed left atrial volume, mL/m^2^	27.69 (20.05, 33.50)	27.64 (20.57, 35.86)	0.623
LV end-diastolic diameter, cm	4.96 (4.50, 5.11)	5.00 (4.79, 5.50)	<0.001 *
Systolic pulmonary artery pressure, mmHg	30.00 (25.34, 36.25)	25.50 (21.00, 36.25)	0.532
TAPSE, mm	22.00 (20.00, 25.00)	24.00 (22.00, 25.25)	0.002 *
LV end-diastolic volume, mL	101.50 (83.75, 117.25)	107.50 (94.75, 120.00)	0.033 *
LV end-systolic volume, mL	52.50 (45.50, 65.25)	55.00 (45.00, 65.00)	0.558
LVEF (%)	46.00 (40.75, 51.00)	50.00 (41.75, 53.00)	0.018 *

E: peak early diastolic mitral flow velocity; A: late transmitral flow velocity; e’: peak early diastolic mitral annulus velocity; s’: peak systolic mitral annulus velocity; RVFW s’: right ventricular free wall peak systolic velocity; LV: left ventricle; TAPSE: tricuspid annular plane systolic excursion; LVEF: left ventricular ejection fraction, IQR: interquartile range. * statistically significant value

**Table 3:** Speckle-tracking echocardiographic parameters of the study population at baseline and at follow-up.

Variable	Initial (n = 60) Median (IQR)	6 Months (n = 60) Median (IQR)	*p* Value
GLS, %	−13.25 (−14.57, −11.55)	−15.60 (−18.25, −12.38)	<0.001 *
MD, ms	53.50 (46.00, 65.25)	55.00 (44.50, 68.25)	0.762
GLS/MD ratio, %/ms	−0.23 (−0.29, −0.19)	−0.27 (−0.41, −0.19)	0.002 *
LS endo, %	−15.15 (−16.50, −13.20)	−18.10 (−20.62, −14.10)	<0.001 *
LS epi, %	−11.00 (−12.72, −9.80)	−13.80 (−16.45, −10.88)	<0.001 *
LS endo/epi ratio	1.33 (1.27, 1.37)	1.30 (1.25, 1.36)	0.496
Mid-CS endo, %	−18.50 (−22.30, −14.05)	−19.60 (−25.00, −13.50)	0.241
Mid-CS epi, %	−7.00 (−10.20, −5.70)	−8.00 (−11.05, −6.40)	0.439
Mid-CS endo/epi ratio	2.45 (1.80, 2.83)	2.23 (1.52, 2.79)	0.277
Base CS, %	−10.80 (−13.40, −6.40)	−11.40 (−14.20, −9.40)	0.004 *
Mid-CS, %	−11.40 (−14.95, −7.40)	−12.30 (−15.10, −9.35)	0.303
Apical CS, %	−11.70 (−16.40, −8.80)	−13.20 (−18.45, −9.50)	0.033 *
Base RS, %	24.83 (14.75, 30.41)	26.83 (15.18, 34.25)	0.399
Mid-RS, %	20.66 (13.33, 32.00)	24.33 (16.10, 40.05)	0.009 *
Apical RS, %	21.00 (13.00, 31.20)	23.00 (16.33, 33.35)	0.507
Twist, °	9.00 (6.17, 13.28)	9.97 (6.50, 14.79)	0.593

GLS: global longitudinal strain; MD: mechanical dispersion; LS endo: segmental strain of endocardium; LS epi: segmental strain of epicardium; Mid-CS: midmyocardial circumferential strain; Mid-CS endo: midmyocardial circumferential strain in the endocardial layer; Mid-CS epi: midmyocardial circumferential strain in the epicardial layer; Mid-RS: midmyocardial radial strain; CS: circumferential strain; RS: radial strain, IQR: interquartile range. * statistically significant value.

**Table 4:** Multivariate linear regression for LS endo at follow-up.

Variable	B	S.E	*p*	95%CI for OR
Thrombolytic therapy	2.853	0.851	0.001	1.184; 4.523
ACEI/ARB	−2.389	0.931	0.013	−4.213; −0.564
LVEF	−0.363	0.055	<0.001	−0.471; −0.255
E/e’ ratio	0.227	0.121	0.066	−0.010; 0.465
Diabetes mellitus	1.765	1.013	0.087	−0.221; 2.847

ACEI: angiotensin-converting enzyme inhibitor; ARB: angiotensin II receptor blocker; LVEF: left ventricular ejection fraction; E: peak early diastolic mitral flow velocity; e’: peak early diastolic mitral annulus velocity.

**Table 5:** General characteristics in cardiac remodelling groups.

Characteristic	No Remodelling (n = 21)	Adverse Remodelling (n = 26)	Reverse Remodelling (n = 13)	*p* Value
Age, years, Median (IQR)	57.00 (50.00, 66.00)	62.00 (53.25, 74.00)	52.00 (42.00, 62.00)	0.064
Male sex	17 (81.0%)	16 (61.5%)	10 (76.9%)	0.304
Current smoker, n (%)	14 (66.7%)	17 (65.4%)	9 (69.2%)	0.972
Systemic hypertension, n (%)	18 (85.7%)	20 (76.9%)	6 (46.2%)	0.035 *
Diabetes mellitus, n (%)	8 (38.1%)	6 (23.1%)	1 (7.7%)	0.132
Hypercholesterolemia, n (%)	21 (100.0%)	26 (100.0%)	13 (100.0%)	0.116
Obesity, n (%)	5 (23.8%)	6 (23.1%)	4 (30.8%)	0.862
Height (cm), Median (IQR)	175.0 (167.0, 178.0)	167.0 (160.0, 175.0)	170.0 (165.00, 174.00)	0.047 *
Weight (kg), Median (IQR)	84.0 (74.0, 90.0)	75.0 (68.0, 86.0)	80.0 (75.0, 81.0)	0.248
Body mass index, kg/m^2^, Median (IQR)	27.45 (25.08, 32.46)	27.05 (25.45, 30.04)	26.40 (25.71, 28.71)	0.769

IQR: interquartile range. * statistically significant value.

**Table 6:** Laboratory and angiographic characteristics in cardiac remodelling groups.

Characteristic	No Remodelling (n = 21)	Adverse Remodelling (n = 26)	Reverse Remodelling (n = 13)	*p* Value
Haemoglobin, g/dL, Median (IQR)	14.60 (13.10, 15.60)	14.70 (13.10, 15.10)	14.20 (13.60, 15.00)	0.972
Blood glucose, mg/dL, Median (IQR)	136.00 (114.00, 161.00)	112 (107.25, 152.75)	109.00 (108, 118)	0.077
White blood count, * 10^3^/µL, Median (IQR)	11.06 (9.33, 12.32)	11.28 (9.44, 14.27)	10.43 (8.60, 13.00)	0.697
Creatinine, mg/dL, Median (IQR)	1.06 (0.97, 1.18)	1.02 (0.91, 1.18)	1.01 (0.90, 1.03)	0.419
LDLc, mg/dL, Median (IQR)	105.0 (79.0, 129.0)	111.50 (96.0, 126.0)	126.0 (102.0, 147.0)	0.132
Triglycerides, mg/dL, Median (IQR)	170.00 (122.0, 205.0)	162.50 (116.0, 209.25)	115.00 (65.0, 128.0)	0.029 *
High-sensitivity cardiac troponin I, ng/L, Median (IQR)	4960 (900, 26000)	10250 (4530, 34576)	1250 (615 11245)	0.096
Thrombolytic therapy, n (%)	10 (47.6%)	11 (42.3%)	4 (30.8%)	0.623
Thrombolytic therapy effectiveness, n (%)Myocardial infarction wall	9 (42.9%)	8 (30.8%)	4 (30.8%)	0.645
Anterior, n (%)	6 (28.6%)	18 (69.2%)	6 (46.2%)	0.020 *
Inferior, n (%)	11 (52.4%)	6 (23.1%)	7 (53.8%)	0.064
Other localisations, n (%)	4 (19.0%)	2 (7.7%)	0 (0.0%)	0.173
LADA, n (%)	17 (81.0%)	20 (76.9%)	9 (69.2%)	0.734
Circumflex artery, n (%)	13 (61.9%)	7 (26.9%)	2 (15.4%)	0.009 *
Intermediate branch, n (%)	3 (14.3%)	1 (3.8%)	0 (0.0%)	0.200
Complete revascularisation, n (%)	11 (52.4%)	15 (57.7%)	10 (76.9%)	0.347
Body surface area, m^2^, Median (IQR)	1.97 (1.85, 2.06)	1.89 (1.74, 1.94)	1.94 (1.85, 1.95)	0.042 *
Beta-blockers, n (%)	19 (90.5%)	20 (76.9%)	9 (69.2%)	0.281
Spironolactone, n (%)	20 (95.2%)	21 (80.8%)	8 (61.5%)	0.047 *
ACEI/ARB	16 (76.2%)	17 (65.4%)	9 (69.2%)	0.722

LDLc: low-density lipoprotein cholesterol; LADA: left anterior descending artery; ACEI: angiotensin-converting enzyme inhibitor; ARB: angiotensin II receptor blocker, IQR: interquartile range. * statistically significant value.

**Table 7:** Echocardiographic characteristics in cardiac remodelling groups.

	No Remodelling (n = 21) Median (IQR)	Adverse Remodelling (n = 26) Median (IQR)	Reverse Remodelling (n = 13) Median (IQR)	*p* Value
E, m/s	0.62 (0.50, 0.77)	0.70 (0.58, 0.89)	0.76 (0.63, 0.82)	0.354
E/A ratio	0.89 (0.72, 1.17)	0.82 (0.63, 1.14)	0.87 (0.80, 1.17)	0.646
Septal e’, m/s	0.07 (0.06, 0.09)	0.07 (0.05, 0.09)	0.08 (0.08, 0.10)	0.101
Septal s’, m/s	0.07 (0.06, 0.09)	0.08 (0.06, 0.08)	0.08 (0.07, 0.09)	0.409
Lateral e’, m/s	0.08 (0.07, 0.10)	0.09 (0.07, 0.10)	0.10 (0.08, 0.13)	0.399
Lateral s’, m/s	0.07 (0.06, 0.09)	0.08 (0.07, 0.09)	0.08 (0.06, 0.09)	0.837
Mean e’, m/s	0.07 (0.07, 0.10)	0.07 (0.07, 0.09)	0.09 (0.08, 0.12)	0.180
E/e’ ratio	7.53 (6.67, 8.33)	9.07 (7.19, 11.81)	7.41 (7.24, 8.44)	0.193
Mean s’, m/s	0.07 (0.07, 0.08)	0.08 (0.07, 0.09)	0.08 (0.07, 0.09)	0.605
E/(e’_s’) ratio	93.24 (78.22, 120.78)	115.28 (87.85, 145.59)	94.79 (82.05, 120.63)	0.428
RVFW s’, m/s	0.14 (0.13, 0.14)	0.15 (0.13, 0.17)	0.14 (0.13, 0.16)	0.149
Left atrial volume, mL	50.00 (36.00, 62.00)	53.50 (44.00, 66.50)	54.00 (42.00, 55.00)	0.478
Indexed left atrial volume, mL/m^2^	25.11 (18.59, 31.07)	30.12 (25.75, 35.64)	27.69 (22.60, 29.67)	0.124
Systolic pulmonary artery pressure, mmHg	30.00 (18.00, 32.00)	32.50 (26.50, 39.00)	30.00 (30.00, 37.00)	0.338
TAPSE	21.00 (20.00, 24.00)	22.00 (21.00, 24.00)	25.00 (22.00, 26.00)	0.079
LV end-diastolic volume, mL	110.0 (98.0, 119.0)	85.50 (73.0, 98.75)	115.00 (110.0, 129.0)	<0.001 *
LV end-systolic volume, mL	60.00 (50.00, 72.00)	46.50 (37.00, 52.75)	61.00 (50.00, 72.00)	0.005 *
LVEF ejection fraction, %	44.00 (40.00, 51.00)	46.00 (41.50, 50.25)	47.00 (41.00, 50.00)	0.959

A: late transmitral flow velocity; E: early diastolic transmitral flow velocity; e’: early mitral annular diastolic velocity; RVFW s’: right ventricular free wall peak systolic velocity; TAPSE: tricuspid annular plane systolic excursion; LV = left ventricle; LVEF: left ventricular ejection fraction; s’ = systolic velocity of mitral annulus, IQR: interquartile range. * statistically significant value.

**Table 8:** Speckle-tracking echocardiography in cardiac remodelling groups.

Variable	No Remodelling (n = 21) Median (IQR)	Adverse Remodelling (n = 26) Median (IQR)	Reverse Remodelling (n = 13) Median (IQR)	*p* Value
GLS, %	−13.40 (−13.95, −11.40)	−13.25 (−14.65, −11.08)	−13.20 (−16.10, −12.50)	0.587
MD, ms	59.00 (50.00, 70.00)	54.00 (48.50, 65.50)	45.00 (45.00, 53.00)	0.073
GLS/MD ratio, %/ms	−0.22 (−0.27, −0.18)	−0.21 (−0.29, −0.18)	−0.29 (−0.36, −0.22)	0.076
LS endo, %	−14.80 (−15.60, −13.20)	−14.55 (−16.88, −12.53)	−15.90 (−18.40, −14.80)	0.251
LS epi, %	−11.00 (−12.30, −9.40)	−11.05 (−12.30, −9.80)	−11.00 (−14.00, −10.50)	0.453
LS endo/epi ratio	1.34 (1.26, 1.37)	1.30 (1.27, 1.36)	1.33 (1.27, 1.39)	0.695
Mid-CS endo, %	−17.70 (−20.30, −13.80)	−18.30 (−24.00, −14.50)	−20.00 (−25.00, −17.90)	0.222
Mid-CS epi, %	−6.80 (−9.70, −5.30)	−8.40 (−10.20, −6.20)	−7.00 (−10.70, −5.40)	0.879
Mid-CS endo/epi ratio	1.85 (1.73, 2.79)	2.32 (1.90, 2.78)	2.56 (2.48, 3.10)	0.295
Base CS, %	−10.80 (−13.50, −6.10)	−10.90 (−13.90, −6.80)	−8.40 (−12.10, −7.20)	0.879
Mid-CS, %	−10.50 (−13.80, −7.00)	−11.50 (−16.70, −8.30)	−11.90 (−14.20, −7.40)	0.456
Apical CS, %	−11.20 (−16.50, −8.40)	−12.00 (−15.40, −9.90)	−14.00 (−18.00, −8.80)	0.671
Base RS, %	21.20 (11.83, 28.83)	24.83 (14.83, 30.33)	29.66 (25.10, 35.00)	0.184
Mid-RS, %	15.00 (9.16, 29.20)	20.66 (16.16, 29.66)	27.16 (26.30, 34.00)	0.064
Apical RS, %	19.50 (9.16, 24.33)	21.83 (14.16, 34.33)	26.83 (18.00, 30.10)	0.123
Twist, °	9.45 (8.00, 14.95)	7.50 (4.64, 10.14)	11.00 (6.15, 14.78)	0.124

GLS: global longitudinal strain; MD: mechanical dispersion; LS endo: segmental strain of endocardium; LS epi: segmental strain of epicardium; Mid-CS: midmyocardial circumferential strain; Mid-CS endo: midmyocardial circumferential strain in the endocardial layer; Mid-CS epi: midmyocardial circumferential strain in the epicardial layer; Mid-RS: midmyocardial radial strain; CS: circumferential strain; RS: radial strain, IQR: interquartile range.

**Table 9:** Multivariate logistic regression for adverse modelling.

Variable	β	S.E	*p*	OR	95%CI for OR
Age	0.095	0.037	0.05	1.100	1.029; 1.176
Triglycerides	0.009	0.004	0.027	1.009	1.001; 1.017
Twist	−0.166	0.074	0.024	0.847	0.733; 0.978
Mid-RS	0.058	0.027	0.032	1.060	1.005; 1.117

Mid-RS: midmyocardial radial strain.

**Table 10:** AUROC of the Twist results for adverse remodelling.

Marker	AUROC	SE AUROC	Lower Limit	Upper Limit	Z Statistic	*p*-Value
Twist	0.64819	0.07233789	0.5064104	0.7899697	2.048581	0.04050307

AUROC: area under the receiver operating characteristic; SE: standard error.

**Table 11:** Cut-off results for Youden index.

Optimal Cut-Off Method: Youden Optimal Cut-Off Point: 11° Performance Measures			
	Values	Lower Limit	Upper Limit
Sensitivity	0.471	0.298	0.649
Specificity	0.769	0.564	0.910
Positive Prediction Value	0.727	0.508	0.857
Negative Prediction Value	0.526	0.346	0.722
Positive Likelihood Ratio	2.039	0.928	4.480
Negative Likelihood Ratio	0.688	0.470	1.007

## Data Availability

The datasets used and analysed during the current study are available from the corresponding author upon reasonable request.
